# The short-chain fatty acid receptor *GPR43* is transcriptionally regulated by XBP1 in human monocytes

**DOI:** 10.1038/srep08134

**Published:** 2015-01-30

**Authors:** Zhiwei Ang, Jun Zhi Er, Jeak Ling Ding

**Affiliations:** 1 Department of Biological Sciences, National University of Singapore, Singapore 117543; 2 NUS graduate School for Integrative Science and Engineering, National University of Singapore, Singapore 117543

## Abstract

G-protein coupled receptor 43 (*GPR43*) recognizes short chain fatty acids and is implicated in obesity, colitis, asthma and arthritis. Here, we present the first full characterization of the *GPR43* promoter and 5′-UTR. 5′-RACE of the *GPR43* transcript identified the transcription start site (TSS) and a 124 bp 5′-UTR followed by a 1335 bp intron upstream of the ATG start codon. The sequence spanning -4560 to +68 bp relative to the *GPR43* TSS was found to contain strong promoter activity, increasing luciferase reporter expression by >100-fold in U937 monocytes. Stepwise deletions further narrowed the putative *GPR43* promoter (−451 to +68). Site-directed mutagenesis identified XBP1 as a core *cis* element, the mutation of which abrogated transcriptional activity. Mutations of predicted CREB, CHOP, NFAT and STAT5 binding sites, partially reduced promoter activity. ChIP assays confirmed the binding of XBP1 to the endogenous *GPR43* promoter. Consistently, *GPR43* expression is reduced in monocytes upon siRNA-knockdown of XBP1, while A549 cells overexpressing XBP1 displayed elevated *GPR43* levels. Based on its ability to activate XBP1, we predicted and confirmed that TNFα induces *GPR43* expression in human monocytes. Altogether, our findings form the basis for strategic modulation of *GPR43* expression, with a view to regulate *GPR43*-associated diseases.

Studies on knockout mice have identified Free Fatty Acid Receptor 2 (*FFAR2* or *GPR43*) as a critical gene in the prevention of obesity, colitis, asthma and arthritis^1^,^2^,^3^,^4^,^5^,^6^,^7^,^8^. *GPR43* is a G protein coupled-receptor that is activated by mid-micromolar concentrations of short-chain fatty acids (SCFAs) - namely acetate, propionate and butyrate. While the liver metabolism of ethanol can generate micromolar concentrations of acetate in the blood, by far the most abundant source of SCFAs in the human body is the colonic lumen, where hundreds of millimolars are continuously being produced during the anaerobic fermentation of dietary fibre by saccarolytic gut bacteria^9^,^10^. These gut SCFAs have been found to beneficially modulate blood glucose and lipid levels, the colonic environment, and immune functions^11^,^12^,^13^. As an SCFA receptor, *GPR43* has already been shown to mediate some of these beneficial effects, with knockout mice studies confirming a role in obesity and inflammation (Table 1).

Consistent with its role as an SCFA receptor, *GPR43* expression is found in cells that are exposed to the highest concentrations of SCFAs. These include the cells of the distal ileum, colon and adipose tissue, with the highest expression found in immune cells such as monocytes and neutrophils^14^,^15^,^16^. In addition, *GPR43* expression appears to be modulated during inflammation since immune challenge by lipopolysaccharide (LPS) or treatment with granulocyte-macrophage colony stimulating factor (GM-CSF) raises *GPR43* transcript levels in human monocytes^17^. This tissue-specificity suggests that *GPR43* expression is tightly regulated and may be important for its function. Indeed, the compelling outcomes of *Gpr43* knockout (Table 1) imply that proper regulation of *GPR43* expression is pertinent to the normal functioning of a range of physiological processes, and consequently, targeting of *GPR43* expression in diseases would provide new therapeutic potential. Thus, details on the factors involved in cell-type specific expression of the *GPR43* gene under pathophysiological conditions remain an unexplored and intriguing area of investigation. Here, we characterized the human *GPR43* gene, identifying the promoter and enhancer sequence elements, as well as the critical transcription factors and signalling pathways that regulate *GPR43* expression.

## Results

### PMA-differentiated U937 monocytes are a suitable model for *GPR43* expression

**Figure 1:**
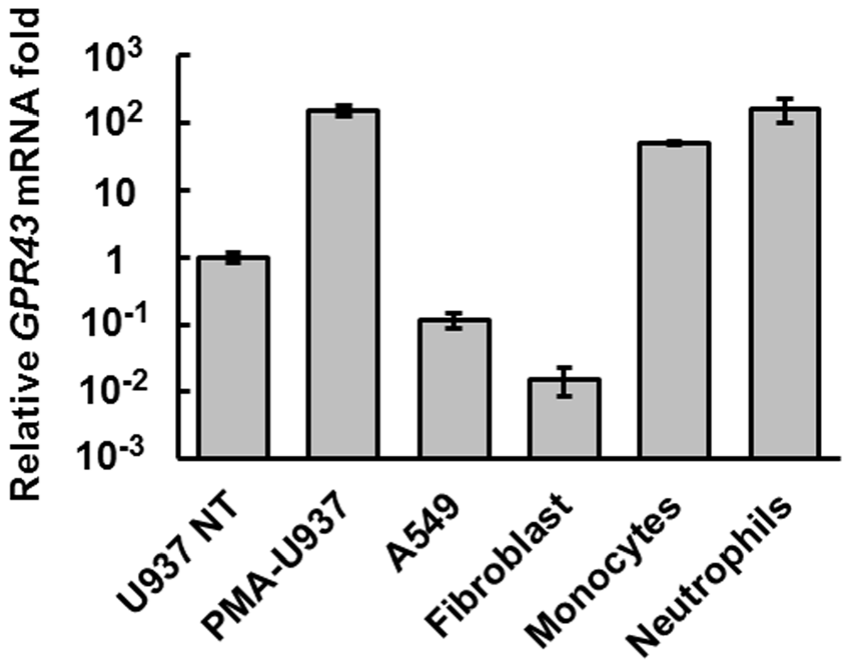
*GPR43* mRNA is up-regulated in primary human monocytes, neutrophils and PMA differentiated U937 cells as determined by quantitative PCR. Results shown are standardized to undifferentiated U937 cells and error bars represent the mean ± s.d. of three independent cell cultures.

To understand the mechanisms underlying the specific expression of *GPR43*, we first confirmed the cell type with the highest level of *GPR43* mRNA. Consistent with previous studies^14^,^15^,^16^, we found human peripheral blood monocytes and neutrophils to express the highest level of *GPR43* mRNA (Fig. 1). PMA-mediated monocytic differentiation of the human promonocytic cell line, U937^18^,^19^, led to a 100-fold increase in the transcription of *GPR43*, yielding mRNA levels that was comparable to the peripheral blood monocytes and neutrophils. Hence, we reasoned that the U937 cell line would be a suitable model to study *GPR43* transcriptional regulation in leukocytes.

### *GPR43* transcription start site is located at 1459 bp upstream of the ATG start codon

**Figure 2:**
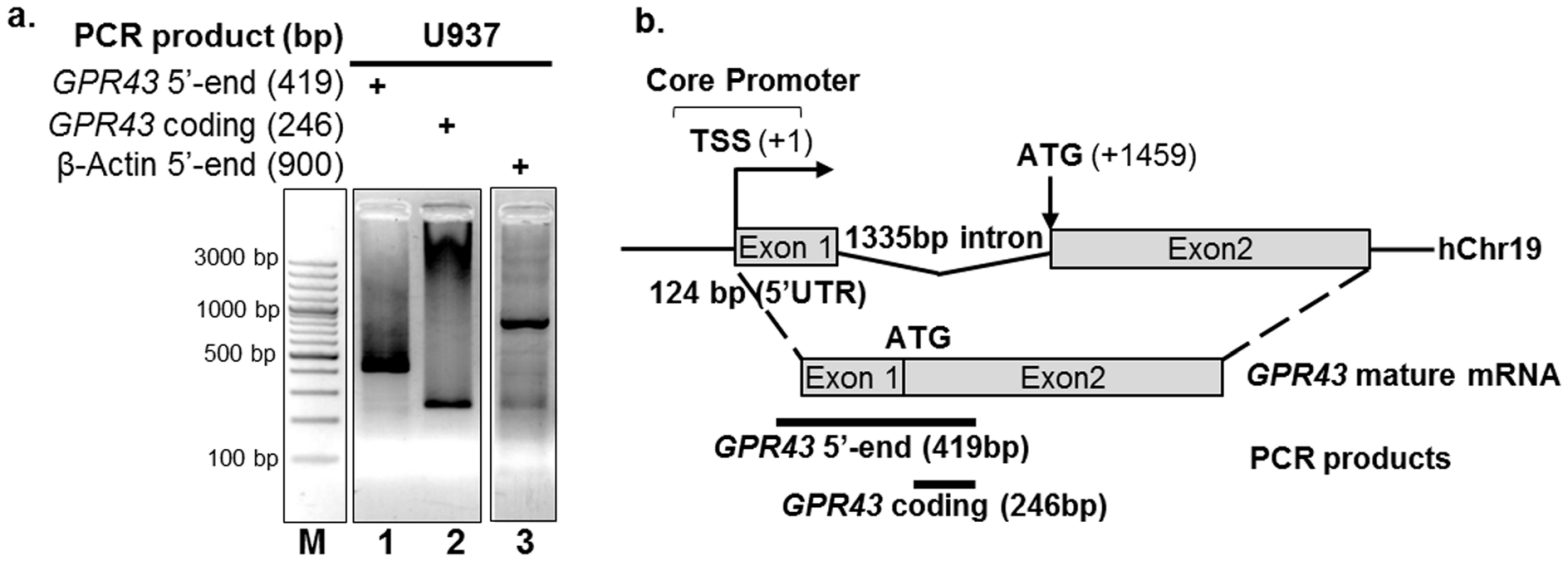
The GPR43 transcription start site is mapped to 1459 bp upstream of the ATG start codon. (a) 5′-RACE of *GPR43* transcripts from U937 cells. Total RNA from U937 cells was extracted and full-length GPR43 mRNA was reverse transcribed using a gene specific primer complementary to its open reading frame. The resulting cDNA was ligated to a known oligonucleotide sequence at the 5′ end. Nested PCR using primers flanking the 5′ end was then performed to procure an approximately 450 bp product encompassing the 5′ untranslated region (5′-UTR) and part of the GPR43 coding sequence (lane 1). As positive controls, amplification of 246 bp of the GPR43 coding region (lane 2), as well as a 900 bp-long 5′ end of the β-actin gene (lane 3) were performed. (b) Schematic mapping the *GPR43* 5′-UTR region and protein coding region on the human chromosome 19. The PCR product from (a, lane 1) was sequenced to reveal a 124 bp long 5′-UTR exon upstream of the *GPR43* ATG start codon.

To define the *GPR43* putative promoter site, we first performed a 5′-RACE of the *GPR43* transcript, generating an approximately 450 bp product which was sequenced to reveal a 124 bp 5′-UTR upstream of the *GPR43* ATG start codon (Fig. 2a). Mapping of this sequence with the human genome database (NCBI Ref Seq: NC_000019.10) revealed the presence of a 1335 bp intron flanked by the *GPR43* 5′-UTR and the start codon (Fig. 2b). Thus, the transcription start site (TSS) is located 1459 bp upstream of the ATG start codon. While it was difficult to judge the presence of other 5′-RACE products (Fig. 2a, Lane 1), we note that a previously reported northern blot analysis (Senga *et al.*, 2003^17^, Fig. 2h), detected two *GPR43* transcripts, with the shorter (by about a few hundred bases) product being many times more abundant than the longer transcript. It is possible that the two transcript sizes described by Senga *et al.*, 2003^17^ are due to different 5′-UTR lengths, which can possibly result from alternate splicing^20^ or promoters^21^. The transcript detected by the 5′ RACE here is likely the shorter, more abundant, transcript. Our attempts to detect the presence of other possible 5′-UTR species via RACE were thus far unsuccessful (data not shown), presumably due to the low abundance. Overall, these results characterize the 5′ region of the human *GPR43* gene and allow the putative core promoter to be identified for subsequent analysis of transcriptional elements.

### The core and proximal promoter of GPR43 are located within −451 to −33 from the TSS

**Figure 3:**
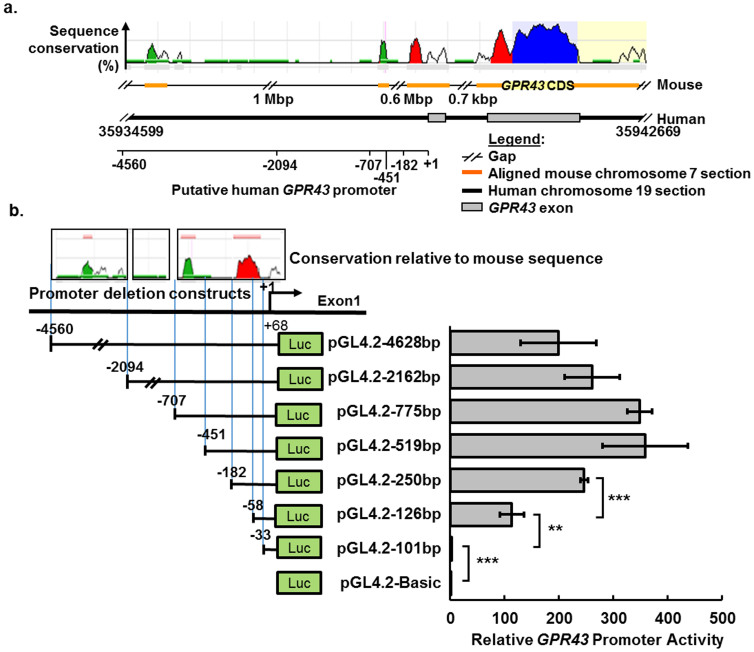
Deletions define a 519-bp putative *GPR43* promoter containing the core and proximal promoter. (a) ECR Browser plot spanning −4560 bp upstream of the transcription start site (+1), the entire *GPR43* gene and 2000 bp after the gene. The plot represents sequence homology of the mouse chromosome 7 (Ref Seq ID: NC_000073.6 ) region flanking the murine *Gpr43* (Entrez Gene ID: 233079) in comparison to the corresponding region on the human chromosome 19 (RefSeq ID: NC_000019.9; positions: 35934599–35942669), which is acting as the baseline. Sequence lengths of >100 bp with 70% sequence conservation are shown as peaks. (b) Luciferase reporter activities of 5′ deletion constructs from a 4628 bp putative *GPR43* promoter in differentiated U937 cells 22 h after transfection. Analysis of the homology with mouse sequence was used to approximate the deletion sites. Results represent the average Firefly luciferase read-outs of three independent transfections (n = 3) normalized to *Renilla* luciferase activity and relative to the basic (empty) luciferase vector, arbitrarily set as 1. Error bars represent the mean ± s.d.. The data shown are representative of three independent experiments. Two tailed Students' T-test was used to determine the statistical significance of the difference between promoter constructs and is annotated as: * <0.05, ** <0.01, and *** <0.001.

Upon identification of the TSS, we cloned a putative promoter region (spanning −4560 to +68 bp relative to the TSS) from primary human monocyte genomic DNA and analyzed its promoter activity using dual luciferase reporter system. This putative promoter contained aligned sequences with greater than 70% sequence identity to the corresponding mouse sequence (Fig. 3a), suggesting conservation with involvement in transcriptional regulation^22^. Indeed, compared to the vector alone, the promoter insert raised the luciferase reporter activity by >100-fold (Fig. 3b). Deletion from positions −4560 to −451 revealed no significant change (p-values > 0.05) in luciferase activity, implying that this region lacks regulatory elements, which is consistent with the general lack of sequence conservation observed (Fig. 3a). Although two short sequence alignments do appear in this region, we note that the corresponding mouse sequences are much further away from the mouse *Gpr43* gene (0.6 Mbp and 1.7 Mbp, respectively), suggesting a lack of involvement in mouse *Gpr43* transcriptional control. Deletion from positions −451 to −182 resulted in a marked drop (p-value = 0.07) in activity while further deletions downstream of position −182 resulted in significant reductions in activity (p values < 0.005), suggesting that the region spanning −451 to −58 might contain crucial activator elements. This is consistent with the observed interspecies sequence conservation (Fig. 3a). Strikingly, the near abrogation of promoter activity upon deletion of −58 to −33 suggests that the core promoter element(s) lies within this 25 bp region.

### XBP1 is part of the core promoter while CREB, CHOP, NFAT and STAT5 act as enhancers

**Figure 4:**
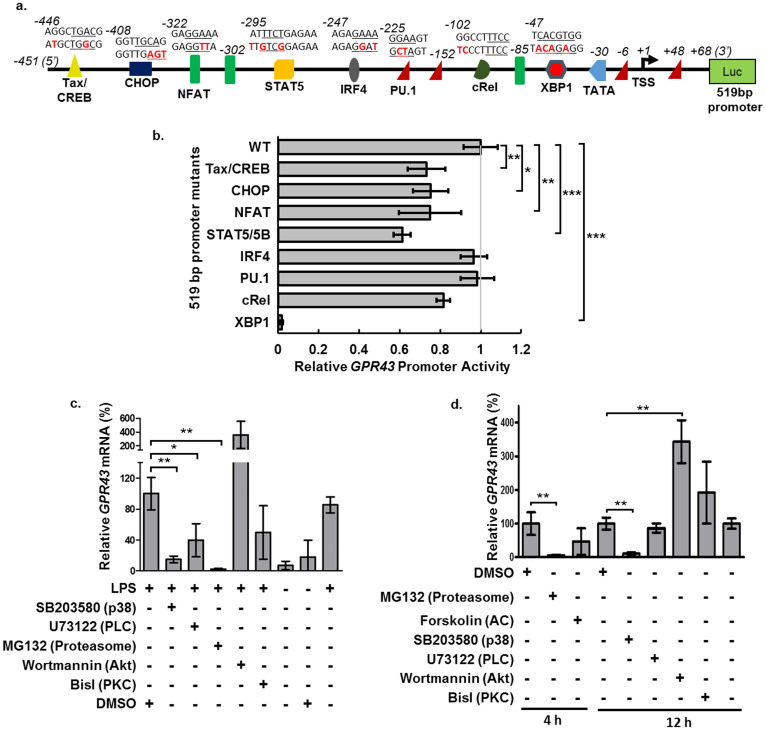
*In silico* predictions, null mutations of the 519 bp promoter region and signalling pathway modulation identify transcription factors (TFs) and pathways regulating *GPR43* expression. (a) Predicted TF binding sites by MatInspector software with corresponding WT and mutant sequences and their relative positions indicated above each TF binding site. The core recognition sequences are underlined while mutated bases are indicated in red. (b) Relative luciferase reporter activities of 519-bp promoter mutated at indicated sites were measured in differentiated U937 monocytes. The multiple binding sites of PU.1 or NFAT residing within the 519 bp promoter were simultaneously mutated in their respective constructs. Wild type (WT) promoter is arbitrarily set to 1 luciferase unit. (c) Quantitative PCR analysis of *GPR43* mRNA levels upon modulation of signalling pathways after 1 h pre-treatment with activators/inhibitors followed by immune challenge for 3 h with 100 ng/mL LPS. (d) Quantitative PCR analysis of *GPR43* mRNA levels upon 4 h treatment of monocytes with inhibitor/activator or 12 h treatment with inhibitor. (c) and (d) Inhibitor/Activator + (Targeted signalling proteins) are shown: SB203580, 10 μM —| p38; U73122, 5 μM —| phospholipase C (PLC); MG132, 10 μM —| Proteasome; Wortmannin, 2 μM —| PI3kinase (PI3K); BisI, 4 μM —| protein kinase C (PKC); Forskolin, 20 μM → Adenylyl cyclase (AC). Expression levels of two reference genes, beta-2-microglobulin (*B2M*) and cyclophilin B (*CYPB*) were also measured and presented as Supplementary Figure S2. All measurements were standardized to the *RPL27* as the reference gene. Experiments were performed in triplicate transfections or treatments, where error bars represent the mean ± s.d. In (b), Dunnett's test (α = 0.05) was performed while in (c) and (d), the two tailed Students' T-test was used to determine statistical significance. p-value * <0.05, ** <0.01, and *** <0.001. The data shown are representative of three independent experiments.

By *in silico* predictions using the MatInspector program^23^, we identified 8 transcription factor (TF) recognition sites with high matrix similarity of ≥90% within the 519 bp putative promoter (Fig. 4a). The null mutations of Tax/CREB, CHOP, NFAT and STAT5/5B resulted in significant losses in activity compared to the wild type (WT) promoter (Fig. 4b), indicating that these TFs may be important enhancers for the control of *GPR43* expression. Conversely, no significant loss in activity was found for mutations in the IRF4, PU.1 and c-Rel recognition sites. Mutation of the XBP1 recognition site resulted in near abrogation of promoter activity (Fig. 4b), hence identifying it as a crucial core *cis* element. Notably, this XBP1 recognition site resides within the 25 bp region that was deleted from the 126 bp promoter (Fig. 3b). XBP1 binding also appears to be required for the low level *GPR43* expression observed in A549 adenocarcinomic human alveolar basal epithelial cells (Supplementary Fig. S1) since the XBP1 null mutation also led to near abrogation in promoter activity in the A549 cells.

### The activities of p38 and PLC are required for *GPR43* transcription

A number of the identified TFs are known to be regulated by MAPK and PLC/PKC pathways^24^,^25^,^26^. To determine if these pathways are involved in the modulation of *GPR43* expression, we blocked the putative pathways with small molecule inhibitors (Fig. 4c, 4d). The relative expression of housekeeping genes was not markedly affected (Supp. Fig. 2). Both SB203580 and U73122, inhibitors of p38 and PLC respectively, attenuated *GPR43* transcription in LPS-challenged monocytes while only SB203580 attenuated *GPR43* transcription in unchallenged monocytes (Fig. 4c, 4d). This suggests that the p38 pathway is required for basal transcription of *GPR43* which is active with or without LPS-challenge while the PLC-PKC pathway is important only during LPS-mediated up-regulation of GPR43. This may be due to p38 pathway activation of XBP1^26^, which is part of the core promoter regulating basal transcription (Fig. 4b); while the PLC-PKC pathway activates NFAT^25^, which we find to act as an enhancer (Fig. 4b). The p38 pathway also activates CHOP^24^. Separately, the proteasome inhibitor, MG132, reduced *GPR43* mRNA levels with or without LPS treatment while Wortmannin, an AKT inhibitor, increased *GPR43* transcript levels. Overall, our findings indicate that activation of the *GPR43* expression is mediated by the p38 and PLC/PKC pathways, and transcriptional regulation may possibly also involve the proteasome degradation and PI3/Akt pathways.

### ChIP analysis confirmed XBP1 binding to *GPR43* promoter *in vivo*

**Figure 5:**
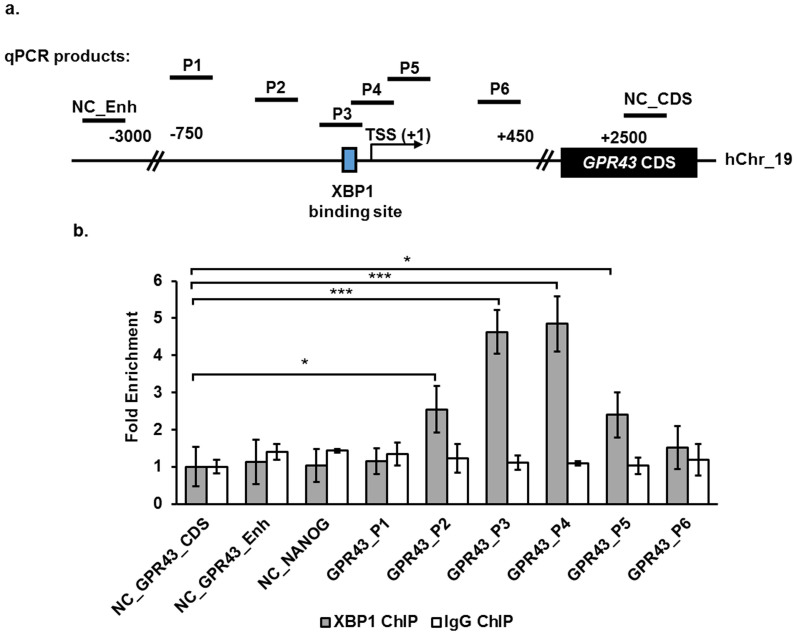
XBP1 binds to the *GPR43* promoter *in vivo*. (a) Schematic showing the relative regions of *GPR43* promoter amplified by quantitative PCR following ChIP assay with anti-XBP1 antibody. P1–P6 spans a region of ~1200 bp surrounding the XBP1 binding site on the *GPR43* promoter. Only promoter regions, P3 and P4 contain the XBP1 binding site. The *GPR43* enhancer region and coding sequence, located more than 3 kb and 2.5 kb up- and downstream of the XBP1 binding site respectively, were also amplified as negative controls. (b) Quantitative PCR analysis of *GPR43* promoter regions following ChIP assay on U937 cells using anti-XBP1 antibody or IgG isotype control antibody. Results are expressed as fold enrichment relative to *GPR43* coding sequence (*GPR43*_CDS) negative control region and error bars represent the mean ± s.d. of three independent pull-down. p-value * <0.05, ** <0.01, and *** <0.001. The data shown are representative of two independent experiments.

Since the XBP1 binding sites were found to be necessary for promoter activity, we investigated XBP1 binding to the endogenous *GPR43* promoter. Chromatin immunoprecipitation (ChIP) was performed with antibodies against XBP1. The *GPR43* promoter XBP1 binding site was significantly enriched in the resulting immunoprecipitate, consistent with strong association of XBP1 to that site (Fig. 5). The pull down appears specific as no significant enrichment was detected for the negative control genomic regions and no *GPR43* promoter enrichment was observed when the isotype control antibody was used.

### XBP1-siRNA knockdown or overexpression alters *GPR43* expression

**Figure 6:**
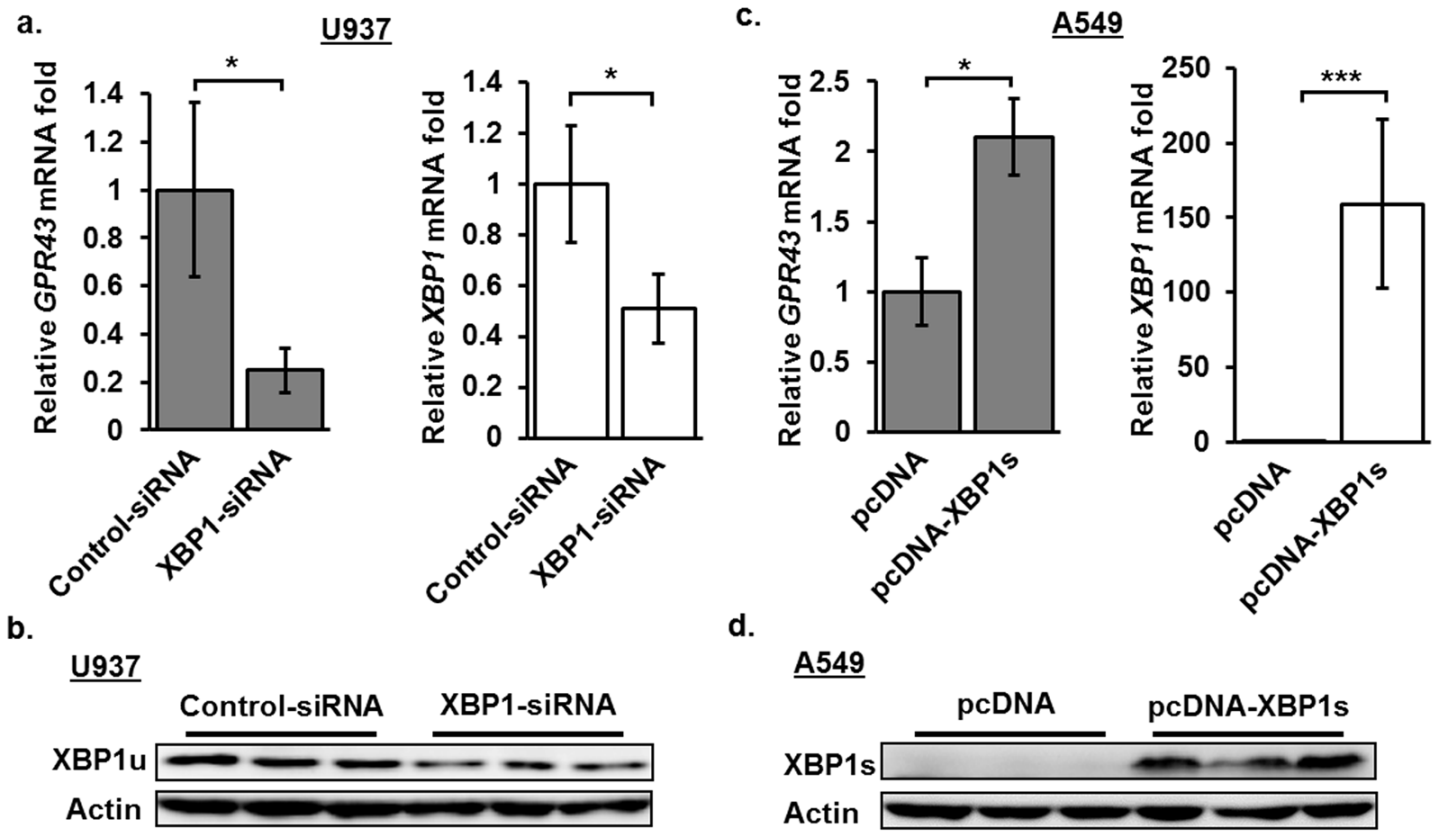
XBP1 is critical for the expression of *GPR43*. (a) Quantitative PCR analysis of *GPR43* and spliced *XBP1* (*XBP1*s) mRNA in XBP1-stable knockdown U937 cells relative to the negative control (non-targeting) siRNA expression plasmid, arbitrarily set as 1. (b) Western blot analysis of unspliced XBP1 (XBP1u) protein levels in XBP1-stable knockdown U937 cells. XBP1s levels were undetectable. (c) Quantitative PCR analysis of *GPR43* and *XBP1* mRNA after 48 h overexpression of XBP1s in A549 cells, relative to the pcDNA empty vector which is set arbitrarily as 1. (d) Western blot analysis of XBP1s protein levels after 48 h overexpression of XBP1s in A549 cells. (b) and (c) Measurements were standardized to *RPL27* as the reference gene. (b) to (d) Results shown are the average of three independent transductions (for stable knockdown) or transfections (for overexpression), with error bars representing the mean ± s.d.. Two tailed Students' T-test was used to calculate statistical significance. p-value * <0.05, *** <0.001.

Next, we sought to confirm that XBP1 activity is required for the endogenous transcription of *GPR43*. Stable knockdown of XBP1 in U937 cells led to a reduction in *GPR43* expression levels relative to the control siRNA (Fig. 6a, b). Conversely, in the A549 adenocarcinomic human alveolar basal epithelial cell line, which expresses low levels of XBP1-dependent *GPR43* mRNA (Fig. 1 and Supplementary Fig. S1), the overexpression of the active spliced form of XBP1 (XBP1s) raised *GPR43* expression levels (Fig. 6c, d). Thus, we have confirmed that XBP1 modulates endogenous *GPR43* expression.

### *GPR43* expression is up-regulated by XBP1 activators

**Figure 7:**
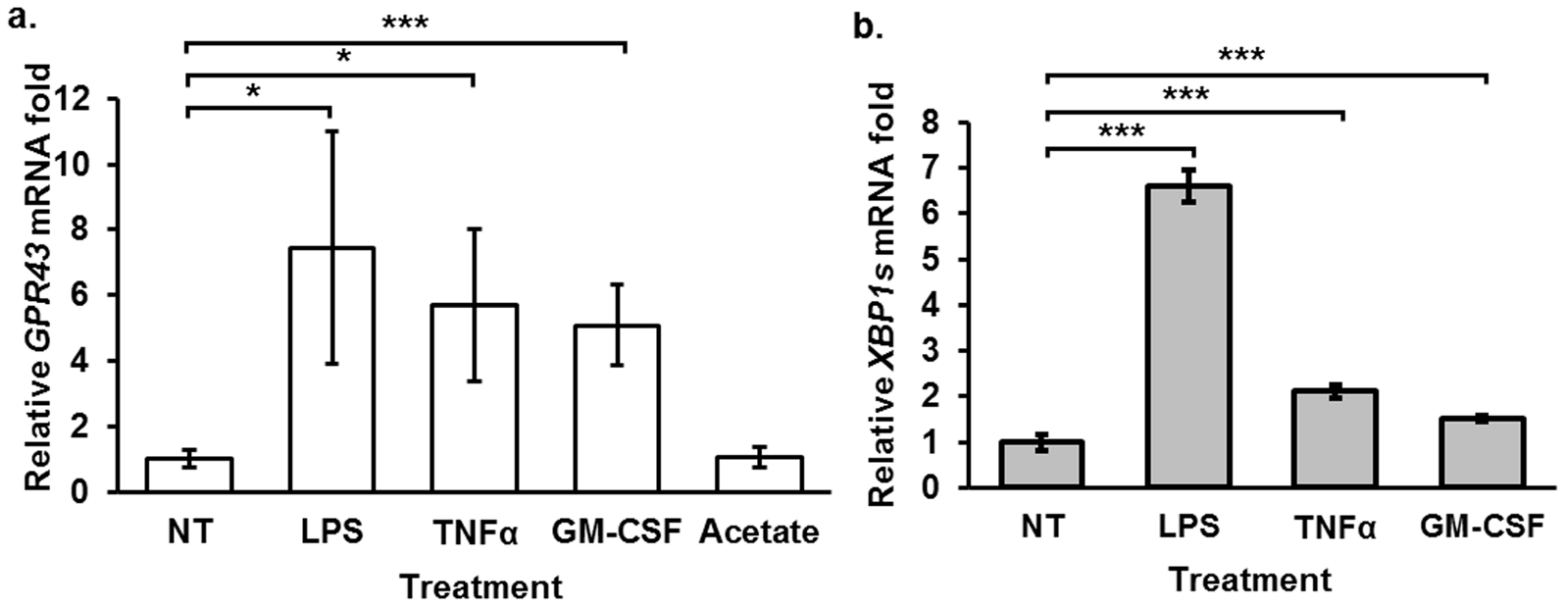
Human monocyte *GPR43* expression is up-regulated by LPS, TNFα and GM-CSF treatment. (a) Quantitative PCR analysis of *GPR43* mRNA and (b) spliced XBP1 (XBP1s) mRNA levels under 3 h treatment with either 100 ng/mL LPS; 10 ng/mL TNFα; 100 ng/mL GM-CSF or 10 mM Acetate (Ac). (a) and (b) The results represent average fold change of the treated samples relative to the non-treated control (NT) and error bars represent the mean ± s.d. of three independent treatment wells. Measurements were standardized to *RPL27* as the reference gene. Two tailed Students' T-test was used to determine the statistical significance of the difference between treated and NT samples. p-value * <0.05, ** <0.01, and *** <0.001. The data shown are representative of at least two independent experiments.

The XBP1-mediated transactivation of *GPR43* prompted us to examine whether *GPR43* expression is increased by known activators of XBP1. LPS is a known activator of XBP1^27^ while GM-CSF is a known positive regulator of dendritic cell development^28^, a process which requires XBP1^29^. We found that LPS treatment increased the *GPR43* transcription by at least 7-fold while GM-CSF raised transcription by 5-fold (Fig. 7a). Both stimuli were previously reported to induce *GPR43* expression via an unknown mechanism^17^. However, our results now revealed this to be attributable to XBP1 activation. Besides LPS, treatment with TNFα, another known activator of XBP1^30^, also up-regulated *GPR43* transcription by 5-fold. Thus, we have demonstrated that pathways leading to the activation of XBP1 consistently up-regulate *GPR43* transcription.

## Discussion

In this study, we identified and characterized the *GPR43* promoter, revealing that XBP1 acts as a core *cis* promoter element while CREB, CHOP, NFAT and STAT5 act as enhancers (Fig. 2, 4a, 4b). Based on its known ability to activate XBP1, we accurately predicted that TNFα up-regulates *GPR43* expression (Fig. 7b). This suggests that other regulators of XBP1 activity may likewise be involved in the regulation of *GPR43* expression. We also showed that *GPR43* expression was altered by inhibition of pathways involved in the activation of CHOP and NFAT (Fig. 4c, 4d). Our identification of the pathways known to activate these *GPR43* promoter elements would facilitate the application of stimuli and conditions to alter *GPR43* expression and its subsequent functions, with a view to therapeutic developments.

The indispensability of XBP1 for *GPR43* promoter activity has important implications since XBP1 is involved in a number of physiological processes and diseases which include B cell development and the unfolded protein response (UPR)^31^. It now appears likely that these physiological processes may engage the expression and function of *GPR43*. Notably, mutations in the XBP1 gene locus of human patients has been associated with increased risk of inflammatory bowel disease (IBD), Crohn's disease and ulcerative colitis, while *Xbp1^−/−^* mice have increased susceptibility to colitis^32^. *Xbp1^−/−^* mice also display impaired glucose and insulin tolerance upon high fat diet-induced obesity^33^. The association of XBP1 with gut inflammation and obesity may be partially attributable to the disrupted expression of *GPR43*, since *Gpr43^−/−^* mice are similarly more susceptible to colitis^1^,^2^,^3^,^4^ as well as exhibiting impaired glucose tolerance and increased obesity^7^,^8^. Considering the implications of XBP1 and *GPR43* in inflammation and obesity, it might be pertinent to further investigate this hypothetical link.

Besides XBP1, the *GPR43* promoter was also found to be up-regulated by Tax/CREB, CHOP, NFAT and STAT5 (Fig. 4). Tax/CREB is a heterodimer consisting of viral oncoprotein, Tax, and the host derived CREB^34^. NFAT and STAT5 have been extensively implicated in immunity and cancer, through regulating cell development, growth and cytokine production^35^,^36^,^37^,^38^. CHOP plays a role in ER stress-induced apoptosis and cytokine production^39^. However, in comparison with XBP1, mutations of the promoter binding sites for the aforementioned four TFs only resulted in relatively modest decrease of ~30–40% in the promoter activity (Fig. 4b). It is hence likely that these TFs are involved in fine-tuning the expression levels of *GPR43* in monocytes, or, they may be more active in up-regulating the gene under the appropriate external stimuli (e.g. cytokine stimulation, ER stress or viral infection). Following the same line of argument, we cannot rule out the potential involvement of other predicted transcription factors, e.g. IRF4, c-Rel (component of NF-κB), and Pu.1 (Fig. 4b), in the expression of *GPR43* under the appropriate immune contexts. This will be a subject of future investigation.

The ability of inflammatory stimuli such as LPS, TNFα and GM-CSF (Fig. 7a) to up-regulate *GPR43* expression infers a number of interesting possibilities. Our findings imply that the positive correlation between *GPR43* expression and inflammation at the fetal cell membranes^40^ or TNFα levels in adipocytes^41^, is likely due to the up-regulation of *GPR43* by the inflammatory stimuli present. Notably, GM-CSF has been found to be beneficial for the treatment of IBD^42^,^43^. Since *GPR43* is also implicated in the etiology of IBD^1^,^2^,^3^,^4^, it would be interesting to investigate whether GM-CSF exerts any beneficial therapeutic effects through modulating *GPR43* expression. In view of the low plasma levels of SCFA (<200 μM)^9^,^10^ and low potencies of the ligands in activating *GPR43* (generally in the mid micromolar range)^14^,^15^,^16^, the higher levels of expression of *GPR43* in the peripheral blood monocytes might thus enhance the potency of SCFAs on the *GPR43*-mediated inflammation.

The increase in *GPR43* expression observed in phosphoinositide-3 kinase (PI3K) inhibition under basal or immune-challenged conditions (Fig. 4c, 4d) suggests that there was a relief of suppression that is mediated by the PI3K/Akt pathway. However, the current information is insufficient to deduce whether this is a direct or indirect effect from the PI3K/Akt signalling. Nevertheless, it should be noted that activation of the PI3K/Akt pathway may lead to the inhibition of p38^44^. Hence, the increase may be due to the recovery of p38, causing an indirect activation of *GPR43* expression.

In addition to characterizing the promoter, we provide the first description of the 5′-UTR of the human *GPR43* gene, identifying an intron between the 5′-UTR and the start codon. Our findings present a new perspective on the human *GPR43* gene, which was initially described as being intronless, downstream of the ATG start codon^45^. Notably, this gene organization is also seen in the bovine *GPR43* gene, the only other *GPR43* homolog which has its 5′-UTR sequenced^46^. Such cross-species similarities in the gene organization and high sequence conservation of the entire core promoter across human and mouse (Fig. 2a) suggests that the same regulatory elements are conserved in the bovine and murine promoters. Indeed, bovine and murine tissue specific expression of *GPR43* have been found to be highly similar to that of the human counterpart^14^,^15^,^16^,^46^. A recent study reported transcriptional activity from a luciferase assay of a 500 bp sequence immediately upstream of the human *GPR43* start codon in mouse RAW264.7 macrophages^47^. Interestingly, these researchers predicted an NF-κB binding site within this 500-bp sequence, although the mutation was reported to cause only 10% reduction in the promoter activity. Perhaps, inclusion of the *GPR43* TSS (located more than 1 kb upstream of the predicted NF-κB binding site as reported by our study) would offer a more complete analysis of the effect of this putative enhancer.

While the findings on the *GPR43* promoter are mostly obtained from human monocytes in this study, it would be interesting to explore if the promoter elements identified, regulate *GPR43* in other cell types. Our findings on A549 cells suggest that this may indeed be the case. In A549 cells, the 519 bp *GPR43* promoter construct up-regulates luciferase expression; an up-regulation that is abrogated by XBP1 null mutation (Supp. Fig. S1). These findings mirror those of U937 cells (Fig. 4b) although differences are also noted. Notably, while a 50% knockdown of *XBP1* mRNA led to an 80% drop in expression of *GPR43* in the U937 cell, a 100-fold increase in *XBP1* mRNA only resulted in a 2-fold increase in A549 expression of *GPR43*. This discrepancy in effect may be due to a variety of factors. XBP1 activity in A549 may already be near saturation and hence further increases in XBP1 expression will not proportionally increase GPR43 expression. Another possibility is that XBP1 may require further activation, such as by the p38 pathway^26^, to become fully activated. Such pathways may be more active in monocytes. The *GPR43* promoter in A549 cells may also be epigenetically silenced through DNA methylation or chromatin remodeling, such that further increases in XBP1 expression may not fully overcome this silencing. Despite these differences, our findings confirm that XBP1 can serve as a regulator involved in controlling *GPR43* expression in other cell types. Notably, both GPR43^15^,^41^,^48^ and XBP1^49^ are found to be up-regulated in adipocytes and adipocyte-derived cell lines, suggesting that XBP1 may also be involved in regulating the expression of *GPR43* in adipocytes, an interaction that may have implications in adipocyte function.

In conclusion, we present the first full characterization of the human *GPR43* promoter, revealing that *GPR43* expression is regulated by XBP1 as a core *cis* element while CREB, CHOP, NFAT and STAT5 act as enhancers. We show that by distinguishing pathways known to activate these *GPR43* promoter elements, it is possible to predict stimuli and conditions that may alter *GPR43* expression and function, thus providing novel drug targets for the treatment of *GPR43*-associated diseases such as obesity, colitis, asthma and arthritis.

## Methods

### Isolation of peripheral blood monocytes, cell culture and differentiation of U937 cells

Peripheral blood mononuclear cells were isolated from the buffy coat of healthy adult donors (National University of Singapore Blood Donation Centre). Ficoll-Paque PREMIUM (GE Healthcare) gradient centrifugation was performed according to the manufacturer's instructions. Briefly, the mononuclear cell layer was isolated and washed with PBS supplemented with 2% FBS (GE healthcare) and 1 mM EDTA, to remove platelets. Monocytes were subsequently purified by negative selection using the Human Monocyte Enrichment Kit (StemCell Technologies) according to the manufacturer's instructions.

Primary monocytes and U937 cell line were cultured in RPMI (Life Technologies) while A549 cells were cultured in DMEM (Life Technologies) supplemented with 10% FBS and 1% (v/v) penicillin and streptomycin (Life Technologies). The cells were grown at 37°C and 5% CO_2_. For differentiation of U937 into monocytes, 5 × 10^5^ cells/mL of pre-differentiated cells were induced with 30 ng/mL phorbol 12-myristate 13-acetate (PMA) (Sigma-Aldrich) for 24 h, before changing to fresh media. The cells were cultured for another 48 h to allow for full differentiation and thereafter used for downstream assays.

The primary neonatal human fibroblasts (Life Technologies) were routinely grown in medium 106 (Life Technologies) before RNA extraction.

### 5′-Rapid Amplification of cDNA Ends (5′ RACE)

Amplification of 5′ cDNA ends of mature GPR43 was performed with the GeneRacer™ kit (Life Technologies, Cat. #L1-502-02) following manufacturer's instructions. Briefly, 5 μg of U937 total RNA was dephosphorylated with Calf Intestinal Phosphatase (CIP) and ligated with a sequence specified RNA oligonucleotide at 37°C for 1 h. Following ligation, the 5′ ends of GPR43 mRNA were reverse transcribed and amplified using nested PCR primers (Supplementary Table S1). The PCR product was then analyzed on a 1.5% agarose gel. The sequence of the GPR43 5′ end was confirmed using Big Dye Terminator cycle sequencing kit and ABI 3100 Genetic Analyser (Life Technologies).

### Construction of *GPR43* promoter and deletion mutants

To generate promoter deletion constructs, primary monocyte genomic DNA was extracted from the interphase and phenol layer using TRIzol reagent (Life Technologies), according to the manufacturer's instructions. To obtain 5′ end promoter deletion products, primers (Supplementary Table S1) flanking the desired promoter regions were used for PCR amplification, using the purified genomic DNA as template. PCR was performed using iProof High Fidelity DNA Polymerase (Bio-rad) under the following parameters: initial denaturation at 98°C; 40 cycles of amplification, denaturation at 98°C for 10 s, annealing at Tm of primer pair +3°C for 30 s, elongation at 72°C for 2.5 min; followed by a final extension at 72°C.

The isolated promoter lengths were separately cloned into pGL4.20 vector (Promega) using standard molecular cloning techniques. The restriction enzymes used were *XhoI* and *HindIII* (Thermo Fisher Scientific). T4 DNA ligase was from Roche. For small-scale purification of plasmids, AxyPrep Plasmid Miniprep kit (Axygen Biosciences) was used. Large-scale purification of plasmids intended for transfection was carried out using PureLink HiPure Plasmid Filter Purification kit (Invitrogen). The full-length sequence of each promoter construct was confirmed by sequencing using Big Dye Terminator cycle sequencing kit and ABI Prism 3100 Genetic Analyzer (Life Technologies).

### *In silico* predictions of transcription factor binding sites and site-directed mutagenesis

Transcription factor binding sites (TFBS) along the delineated promoter regions were identified using the MatInspector programme available online at http://www.genomatix.de/23. Analysis of the sequences was performed using the MatInspector TFBS, weight matrix library version 9.0. Only predicted TFBS with a core matrix similarity of ≥0.95 and an overall matrix similarity of ≥0.90, were considered significant.

Promoters containing the specific mutated TFBS were synthesized by Integrated DNA technologies. The mutated promoters were cloned into pGL4.20 vector (Promega) and confirmed by sequencing as described above.

### Transient transfection and luciferase reporter assay

The full-length promoter, deletion and mutant constructs were transiently transfected into differentiated U937 cells at 48 h after removal of PMA, with X-tremeGENE HP DNA Transfection Reagent (Roche) according to the manufacturer's guidelines. Promoter-pGL4.20 vector constructs and pRL-CMV control *Renilla* luciferase vector (Promega) were transfected in the molar ratio of 10:1.

*Renilla* luciferase reporter activities were assessed using the Dual Luciferase Reporter Assay System (Promega) 22 h after transfection. Luminescence was detected using the Glomax 20/20 Luminometer (Promega), and the resulting measurements from the Firefly luciferase were normalized to the *Renilla* luciferase. Relative light units were calculated using readouts from the pGL4.20-Basic (promoter-less) vector or the wild type promoter as baseline for deletion and mutation constructs, respectively.

### Treatment of cells with inflammatory stimuli and pathway activation/inhibition

For stimulation of cells, *Escherichia coli* 055:B5 LPS and sodium acetate were purchased from Sigma-Aldrich while GM-CSF and TNFα were from Life Technologies. Purified human monocytes were seeded at a density of 4 × 10^6^ cells/mL in 24-well plates and cultured in the presence of either LPS (100 ng/mL), PMA (30 ng/mL), TNFα (10 ng/mL), GM-CSF (100 ng/mL) or sodium acetate (10 mM) for 3 h before RNA extraction with TRIzol reagent (Life Technologies).

For modulation of signalling pathways, p38 inhibitor *SB203580*, PKC inhibitor *Bisindolylmaleimide I* (*BisI*) (all from Cell Signalling Technology), PLC inhibitor *U73122*, adenylate cyclase activator *Forskolin* (Tocris Biosciences), proteasome inhibitor *MG132* and PI3K inhibitor *Wortmannin* (Sigma) were obtained.

Purified human monocytes were pre-treated for 1 h in either 20 μM DMSO (Merck) as vehicle control, SB203580 (10 μM), U73122 (5 μM), MG132 (10 μM), Wortmannin (2 μM) or BisI (4 μM). Cells were then challenged in 100 ng/mL LPS for 3 h before mRNA was extracted for analysis.

To identify regulatory pathways involved in the basal regulation of gene expression, the same conditions above were applied except that replicate wells of cells treated with MG132 (10 μM) and Forskolin (20 μM) were lysed and analyzed for mRNA expression after 4 h without LPS treatment. For treatment with other inhibitors, monocytes were lysed for RNA extraction and analyzed after 12 h in culture.

### Quantitative PCR (qPCR)

Total RNA was isolated using the TRIzol reagent according to the manufacturer's instructions and the procedure was repeated to ensure thorough removal of genomic DNA. cDNA synthesis of the extracted RNA was performed using Superscript III First Strand Synthesis kit (Life Technologies). Quantitative PCR was performed using GoTaq qPCR Master Mix (Promega) and forward and reverse primers (Supplementary Table S1) with LightCycler 480 system (Roche). Spliced *XBP1* was detected using the Taqman gene expression assay (Life Technologies, Hs03929085_g1) and Light Cycler Probes Master (Roche). To obtain relative target mRNA folds, cycle thresholds (Ct) were normalized to the Ct of Glyceraldehyde 3-phosphate dehydrogenase (*GAPDH*) or Ribosomal protein L27 (*RPL27*) reference genes and expressed as fold-change by the 2^−▵▵Ct^ method^50^.

### Western Blotting

Cell lysates were separated by SDS-PAGE and transferred onto PVDF membranes (Bio-rad). The blots were then probed with antibodies against XBP1 (Abcam, ab37152), or β-actin (Sigma, a2066) followed by the corresponding secondary antibodies according to respective manufacturer's instructions. Following incubation with WesternBright ECL chemiluminescent substrate (Advansta), chemiluminescent signals was detected with an ImageQuant™ LAS 4000 mini (GE Healthcare).

### ChIP assay

ChIP assay was performed as described previously^51^. Differentiated U937 cells were cross-linked with 1% formaldehyde for 10 min at room temperature and then quenched with 0.2 M of glycine. Chromatin extracts from lysed cells were sonicated to an average DNA fragment length of 350 bp and immunoprecipitated with Santa Cruz Biotechnology antibodies anti-XBP1 (sc-7160) or IgG isotype control (sc-2027). Following de-crosslinking, quantitative PCR was performed with multiple ChIP primers (Supplementary Table S1) to quantitate the relative occupancy of the *GPR43* promoter region. Fold enrichment was obtained by normalizing the amount of precipitated DNA to that of the input sample and then expressed relative to the *GPR43* negative control coding region.

### siRNA knockdown and XBP1s overexpression

For stable knockdown of XBP1, siRNA sequences Sense: 5′-AAAGACAGCAAGTGGTAGATTTA-3′; Anti-sense: 5′-AAAATAAATCTACCACTTGCTGT-3′^52^ was cloned into pFIV-H1/U6 vector (System Biosciences), while a non-targeting sequence was cloned as the negative control vector. The resulting siRNA vector construct or pFIV-H1/U6-copGFP vector (System Biosystems), which contains CopepodGFP coding sequence in replacement of the puromycin selection marker, was co-transfected with lentiviral packaging vectors pFIV-34N and pVSV-G into HEK-293T cells with TurboFect transfection reagent (Thermo Scientific). Culture supernatant was collected after 48 h and used to resuspend U937 cells, with the addition of polybrene to a final concentration of 8 μg/mL. The resulting suspensions were then centrifuged at 1000 g for 2 h at 37°C, after which the transduced cells were re-suspended in 2 mL of fresh complete RPMI media and seeded into 6 well plates. Successfully transduced cells were selected with 1 μg/mL puromycin (Life technologies) in culture medium 48 h after transduction, for 1.5 weeks.

For *XBP1s* overexpression, *XBP1s* coding sequence (NM_001079539.1) was cloned into pcDNA3.1A (Invitrogen) and transfected into A549 cells using X-tremeGENE HP DNA Transfection Reagent (Roche) according to the manufacturer's instructions.

## Author Contributions

A.Z.W. and E.J.Z. conceived, designed, and performed the experiments and analyzed the data with intellectual input from J.L.D. J.L.D. provided overall coordination and supervision of the study. A.Z.W., E.J.Z. and J.L.D. wrote the manuscript.

## Supplementary Material

Supplementary Information Supplementary Information

## Figures and Tables

**Table 1:** Known phenotypes of *GPR43* knockout mice

Publication:	*Gpr43* knockout mice phenotype:
Maslowski *et al.* 2009^1^	Exacerbated colitis, arthritis and asthma
	Reduced neutrophil recruitment
Sina *et al.* 2009^2^	Reduced colitis
	Increased neutrophil recruitment
Kim *et al.* 2013^3^	Reduced colitis
	Reduced ERK and p38 activation in epithelial cells
Smith *et al.* 2013^4^	Exacerbated colitis
	Reduced Treg cell count
Ge *et al.* 2008^53^	Increased lipolysis and plasma free fatty acids
Bjursell *et al.* 2011^6^	Improved glucose control and reduced body fat mass on a high fat diet
Tolhurst *et al.* 2012^7^	Impaired glucagon-like peptide-1 secretion and glucose tolerance
Kimura *et al.* 2013^8^	Increased fat accumulation and obesity on a normal diet
