# Supplementary material for: The short-chain fatty acid receptor GPR43 is transcriptionally regulated by XBP1 in human monocytes
Source: Sci Rep. 2015 Jan 30;5:8134. doi: 10.1038/srep08134 (PMC4311239; doi:10.1038/srep08134)
Supplement: Supplementary Information [file srep08134-s1.doc]

**The short-chain fatty acid receptor *GPR43* is transcriptionally regulated by XBP1 in human monocytes**

Zhiwei Ang^1,2,ξ^ , Jun Zhi Er^1,2,ξ^, and Jeak Ling Ding^1,2,*^

**SUPPLEMENTARY INFORMATION**

**Table S1: List of primers used**

| **Name** | **Sequence (5’ > 3’)** | **Purpose** |
| --- | --- | --- |
| 5’ RACE | | |
| 5’-GR | CGACTGGAGCACGAGGACACTGA | Forward primer |
| 5’-GRN | GGACACTGACATGGACTGAAGGAGTA | Nested forward primer |
| 317-5 | TCGATGCTGATGCCCGCCAG | Reverse primer |
| 265-5 | AGCCAAAACTCGTGAGGGCG | Nested reverse primer |
| 394R-5 | ccagagctgcaatcactcca | Gpr43 gene specific reverse transcription |
| 19-3 | AGCTCCTTGATCCTCATGGCTTACA | Gpr43 coding sequence forward primer |
| Promoter Cloning | | |
| 442-3 | CCCGCTCGAGAAGACCACAACAAAGGCCGGGTACGGTGGTCC | Forward primer for 4628 bp promoter |
| 2908-3 | CCCGCTCGAGAGGCACAGAACCCAGATTAGGGTGGACTGAGGGGCG | Forward primer for 2162 bp promoter |
| 5429-5 | CACAGGCCTTCACTGGCCCTTGAGCGTGGCAT | Reverse primer for 4628 bp and 2162 bp promoter |
| 3744-3 | CCCGCTCGAGTTTCAGCTGAGACGGGCAAA | Forward primer for 775 bp promoter |
| 257-3 | CCCGCTCGAGGAGGCTGACGCAGGAGAATC | Forward primer for 519 bp promoter |
| 650-3 | CCCGCTCGAG CTTTCTCTGGTCACGTGGCTG | Forward primer for 126 bp promoter |
| 675-3 | CCCGCTCGAGTAGTATAAATGCTTACTACCAGCCA | Forward primer for 101 bp promoter |
| 769R-5 | CCCAAGCTTCCAGGAGAGAGGAACAGAGC | Reverse primer for 775 bp, 519 bp, 126 bp, 101 bp promoter |
| Sequencing | | |
| RVP-3 | TAGCAAAATAGGCTGTCCC | pGL4.20 forward sequencing primer |
| 96R-5 | GGCTTTACCAACAGTACCGGA | pGL4.20 reverse sequencing primer |
| 499-3 | ACACTGCCAGGTATTTGCCCAACAGCACTGAAAACA | Forward primer for 4628 bp promoter sequencing |
| 2680-3 | CGGGGATGAGTAGGGGAATGGTGAGCTAGCAAAGGG | Forward primer for 4628 bp promoter sequencing |
| 1268-5 | CCAGCCCCTGCCTGGTATGTCTTTATTTC | Reverse primer for 4628 bp promoter sequencing |
| Quantitative PCR | | |
| GPR43 | F: GTAGCTAACACAAGTCCAGTCCT  R: CTAGGTGTTGCTTTGAAGCTTGT | qRT-PCR of *GPR43* |
| GAPDH | F: CGTCTTCACCACCATGGAGA  R: CGGCCATCACGCCACAGTTT | qRT-PCR of *GAPDH* |
| XBP1 | F: AACCATTCTTGGGAGGACACTTT  R: TCCAGGCAGTGTAATAGTCAAGG | qRT-PCR of *XBP1* |
| B2M | F: TGAGTATGCCTGCCGTGTGAAC  R: TGCTGCTTACATGTCTCGATCCC | qRT-PCR of *B2M* |
| RPL27 | F: ATCGCCAAGAGATCAAAGATAA  R: TCTGAAGACATCCTTATTGACG | qRT-PCR of *RPL27* |
| CYPB | F: TGGCACAGGAGGAAAGAGCA  R: AAAGGGCTTCTCCACCTCGATC | qRT-PCR of *CYPB* |
| ChIP | | |
| NC_Gpr43_CDS | F: TTCAGAAATCCTTAGACCCAGCC  R: ACTTCTTCGTGCATCTCTGACTT | Negative control ChIP primer |
| NC_Gpr43_Enh | F: GAAGCAGATGGATGAGAGGAAGT  R: GCAGTCTGATGTACTCCCCAAAT | Negative control ChIP primer |
| Gpr43_P1 | F: TGGATTTGAGCCCATATCTGCAT  R: CAACACATTTCTTTTGCCCGTCT | *GPR43* promoter region |
| Gpr43_P2 | F: GAGCAGAATGACAGAAGAAACTGC  R: CTCTCCCTAGAGAATCCTCACTCT | *GPR43* promoter region |
| Gpr43_P3 | F: GAGAGAATAAAGATGCTGGGCCT  R: AACCTGGCTGGTAGTAAGCATTT | *GPR43* promoter region |
| Gpr43_P4 | F: ACGTGGCTGGTGCTAGTATAAAT  R: GCTTCTTGGGATTGGTTCTGAAG | *GPR43* promoter region |
| Gpr43_P5 | F: CCAATCCCAAGAAGCCACCTATC  R: CTCCTGTCTCTGGGTATGAGTCT | *GPR43* promoter region |
| Gpr43_P6 | F: GAAACTGAGTTACCCCGTGAAGA  R: GGCTTGTCCCTAGAAGACCTTAG | *GPR43* promoter region |
| Nc_Nanog | F: GGGCTGCACTGCACATTGAC  R: GGCACGCTTGCGTATGTCTG | Negative control ChIP primer (different locus) |


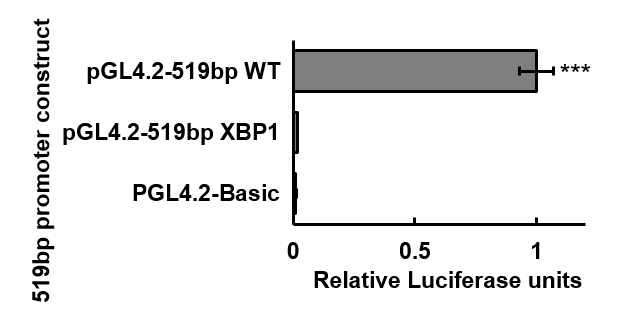


**Supplementary Figure S1. *GPR43* 519 bp promoter activity in A549 cells.** Luciferase reporter activities of the 519 bp putative wild type (WT) *GPR43* promoter or mutated at the XBP1 binding site in A549 cells 22 h after transfection. Results represent the average Firefly luciferase read-outs of three independent transfections (n=3) normalized to *Renilla* luciferase activity and relative to the basic (empty) luciferase vector, arbitrarily set as 1. Error bars represent the mean ± s.d. Two tailed Students’ T-test was used to determine the statistical significance of the difference between promoter constructs and is annotated as: * < 0.05, ** < 0.01, and *** < 0.001.


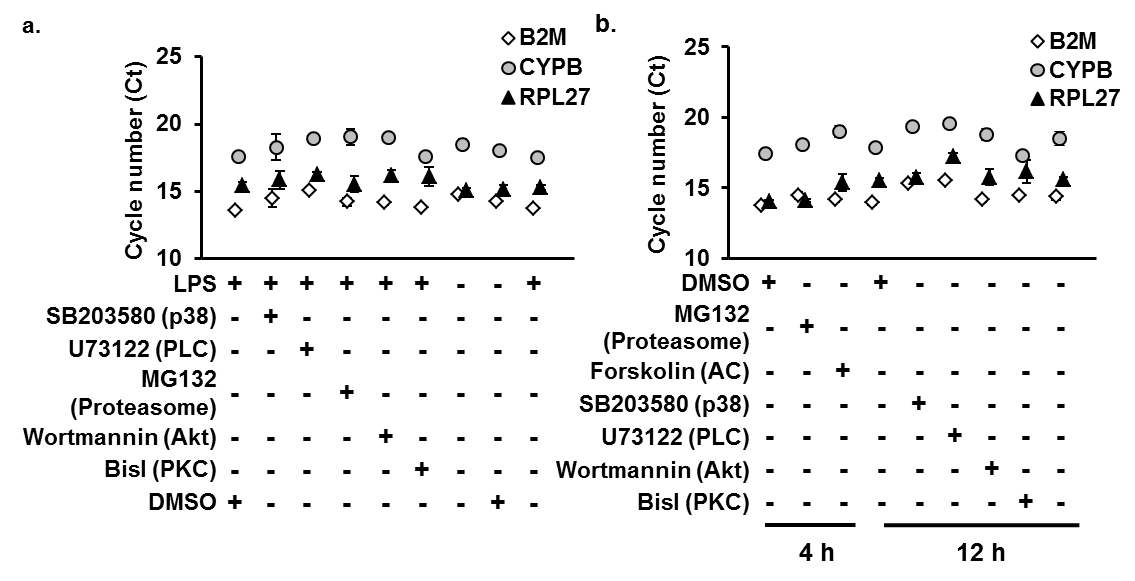


**Supplementary Figure S2. Expression levels of *B2M,* *CYPB* and *RPL27* upon signalling pathway modulation. (a)** Quantitative PCR analysis of *B2M, CYPB*  and *RPL27* mRNA levels upon modulation of signalling pathways after 1 h pre-treatment with activators / inhibitors followed by immune challenge with 3 h (100 ng/mL) LPS. **(b)** Quantitative PCR analysis of *B2M* and *CYPB* mRNA levels upon 4 h treatment of monocytes with inhibitor / activator or 12 h treatment with inhibitor. **(a and b)** Inhibitor / Activator + (Targeted signalling proteins) are shown: SB203580, 10 µM **|** p38; U73122, 5 µM **|** phospholipase C (PLC); MG132, 10 µM **|** Proteasome; Wortmannin, 2 µM **|** PI3kinase (PI3K); BisI, 4 µM **|** protein kinase C (PKC); Forskolin, 20 µM 🡪 Adenylyl cyclase (AC). All measurements were standardized to *RPL27* as the reference gene. Experiments were performed in triplicate treatments, where error bars represent the mean ± s.d.
